# Sleep-related hypermotor epilepsy: Long-term outcome in a large cohort

**DOI:** 10.1212/WNL.0000000000003459

**Published:** 2017-01-03

**Authors:** Laura Licchetta, Francesca Bisulli, Luca Vignatelli, Corrado Zenesini, Lidia Di Vito, Barbara Mostacci, Claudia Rinaldi, Irene Trippi, Ilaria Naldi, Giuseppe Plazzi, Federica Provini, Paolo Tinuper

**Affiliations:** From IRCCS Istituto delle Scienze Neurologiche di Bologna (L.L., F.B., L.V., C.Z., B.M., G.P., F.P., P.T.) and Department of Biomedical and Neuromotor Sciences (L.L., F.B., L.D.V., C.R., I.T., I.N., G.P., F.P., P.T.), University of Bologna, Italy.

## Abstract

**Objective:**

To assess the long-term outcome of sleep-related hypermotor epilepsy (SHE).

**Methods:**

We retrospectively reconstructed a representative cohort of patients diagnosed with SHE according to international diagnostic criteria, sleep-related seizures ≥75% and follow-up ≥5 years. Terminal remission (TR) was defined as a period of ≥5 consecutive years of seizure freedom at the last follow-up. We used Kaplan-Meier estimates to calculate the cumulative time-dependent probability of TR and to generate survival curves. Univariate and multivariate Cox regression analyses were performed.

**Results:**

We included 139 patients with a 16-year median follow-up (2,414 person-years). The mean age at onset was 13 ± 10 years. SHE was sporadic in 86% of cases and familial in 14%; 16% of patients had underlying brain abnormalities. Forty-five percent of patients had at least 1 seizure in wakefulness lifetime and 55% had seizures only in sleep (typical SHE). At the last assessment, 31 patients achieved TR (TR group, 22.3%), while 108 (NTR group, 77.7%) still had seizures or had been in remission for <5 years. The cumulative TR rate was 20.4%, 23.5%, and 28.4% by 10, 20, and 30 years from inclusion. At univariate analysis, any underlying brain disorder (any combination of intellectual disability, perinatal insult, pathologic neurologic examination, and brain structural abnormalities) and seizures in wakefulness were more frequent among the NTR group (*p* = 0.028; *p* = 0.043). Absence of any underlying brain disorder (hazard ratio 4.21, 95% confidence interval 1.26–14.05, *p* = 0.020) and typical SHE (hazard ratio 2.76, 95% confidence interval 1.31–5.85, *p* = 0.008) were associated with TR.

**Conclusions:**

Our data show a poor prognosis of SHE after a long-term follow-up. Its outcome is primarily a function of the underlying etiology.

Sleep-related hypermotor epilepsy (SHE), previously known as nocturnal frontal lobe epilepsy (NFLE), is a focal epilepsy characterized by hypermotor seizures occurring predominantly in clusters during non-REM sleep. The clinical features and diagnostic criteria of SHE were recently revised during an international consensus conference (figure e-1 at Neurology.org).^1^

Etiologic factors underlying SHE encompass genetic and structural causes, without significant differences in clinical features between sporadic cases and the familial form with autosomal dominant inheritance (ADSHE; previously autosomal dominant nocturnal frontal lobe epilepsy).^1^

To date, studies addressing the long-term outcome of the whole spectrum of SHE syndrome are lacking and the prognostic data are contradictory and are derived from noncohort studies or from subpopulations of patients with SHE. ADSHE has been proposed as a paradigm of a distinct benign focal epilepsy syndrome occurring in patients of normal intelligence,^2^ despite several reports of intellectual disability (ID)/psychiatric disorders and early-onset refractory epilepsy associated with distinctive mutations in the nicotinic acetylcholine receptor subunit genes^3^ and with specific genes.^4^ The largest case series on sporadic SHE suggested an overall drug resistance rate of 30%, comparable to other focal epilepsies.^5^ The few cohort studies available focused on the surgical outcome of patients with refractory SHE.^6,7^

We aimed to assess the long-term outcome in terms of 5-year seizure freedom (SF) rate (terminal remission [TR]) in a large, representative cohort of consecutive patients diagnosed with SHE according to the new shared inclusion criteria.^1^ In addition, we analyzed clinical predictors for TR.

## METHODS

This study has a cohort design and is reported following the STROBE (Strengthening the Reporting of Observational Studies in Epidemiology) guidelines.^8^

### Participants, setting, and eligibility criteria.

The study was conducted at the IRCCS Institute of Neurologic Sciences of Bologna between September 2012 and October 2013.

**Figure 1:**
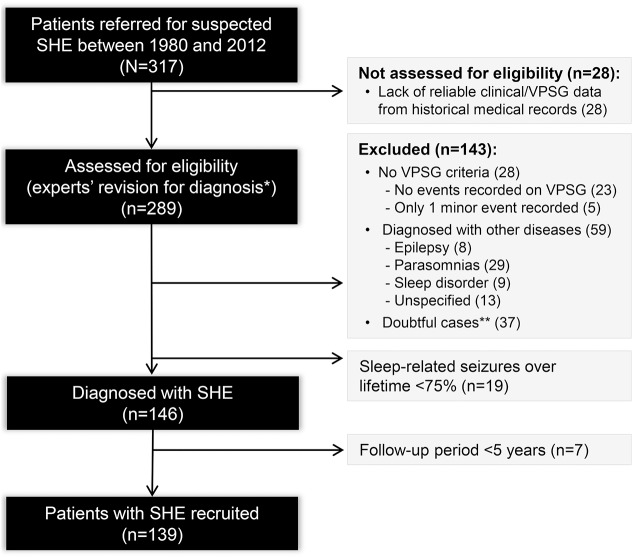
Flow diagram of patient recruitment SHE = sleep-related hypermotor epilepsy. *The final diagnosis was confirmed by 3 experts in sleep medicine and epileptology (P.T., F.P., F.B.). The final agreement required was 100%; otherwise, cases were considered doubtful and excluded. **All cases with video-polysomnographic (VPSG) recording of motor events of uncertain nature/not stereotyped (n = 34) or for which there was no agreement among the experts (n = 3).

An initial pool of patients with suspected SHE referred to our institute from 1980 was reconstructed by reviewing medical and video-polysomnographic records from the archive and the databases of the epilepsy and sleep centers (figure 1).

All patients had video-EEG documentation of at least 1 sleep-related hypermotor event. Diagnosis of SHE was based on level of certainty according to the new diagnostic criteria (figure e-1).^1^

### Inclusion criteria.

We included patients with video-documented (clinical) or video-EEG–documented (confirmed) diagnosis of SHE and a follow-up of ≥5 years.

Because current diagnostic criteria do not specify the all-life proportion of seizures arising from sleep with respect to wakefulness,^1^ we labeled the frequency of sleep-related seizure according to the following categories: (1) 100% sleep-related seizures, i.e., no seizures during wakefulness lifetime (these patients were labeled as having a typical SHE pattern); (2) sleep-related seizures >75%, i.e., the frequency of seizures during sleep largely predominated seizures in wakefulness lifetime; and (3) sleep-related seizures <75%, i.e., the proportion of seizures from sleep slightly exceeded seizures during wakefulness; these patients were excluded from the study.

### Data collection.

A medical chart recording clinical and instrumental updates at every control visit was reviewed for each patient.

The last assessment was conducted by a direct visit by October 2013. Patients unable to attend a control visit by 1 year after the beginning of recruitment underwent a semistructured telephone interview. We focused principally on SF, ascertained as seizure frequency over the preceding 5 years, by reviewing a seizure diary. All data were collected in an ad hoc database. The e-appendix provides details on database items, data collection, and statistics.

### Outcomes and prognostic factors.

The primary endpoint was TR, a period of at least 5 consecutive years without seizures at the last follow-up, regardless of treatment status.^9^ On the basis of the primary end-point, we distinguished between the TR group, made up of patients with SF for ≥5 years at the last visit, and the NTR group, made up of patients not attaining TR.

We observed 3 patterns of seizure occurrence over time: a remitting pattern in patients who achieved TR at some point of their disease history without any relapse, a continuous pattern in patients having no remission at all after epilepsy onset, and an intermittent pattern in patients in whom the epilepsy course showed some relapsing periods. We defined relapse as the occurrence of any seizure after a 5-year remission had been achieved during the epilepsy course.^10^

Potential prognostic factors examined are reported in table 1. The additional variable of any underlying brain disorder was defined as at least one of the following causative factors, possibly in combination: ID/borderline IQ, personal history of perinatal insult, pathologic neurologic examination (NE), and brain abnormalities.

**Table 1:**
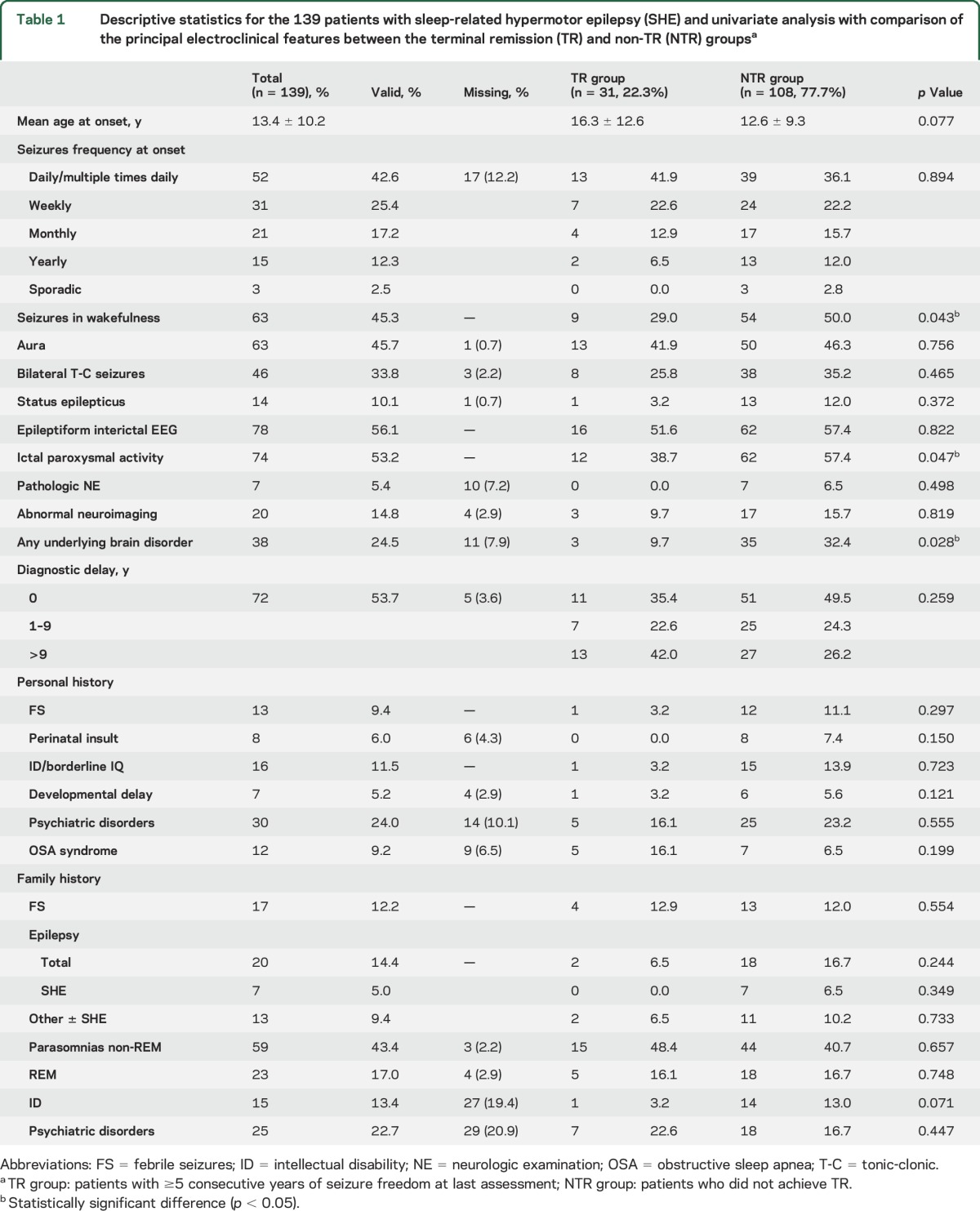
Descriptive statistics for the 139 patients with sleep-related hypermotor epilepsy (SHE) and univariate analysis with comparison of the principal electroclinical features between the terminal remission (TR) and non-TR (NTR) groups^a^

We referred to the International League Against Epilepsy guidelines of drug resistance,^11^ ascribing the degree of pharmacoresistance according to Perucca, 1998.^12^

Diagnosis was dated from either video-polysomnographic recording of SHE seizures or diagnosis of focal epilepsy, even if not better specified, followed by prescription of appropriate antiepileptic drugs (AEDs). In patients whose diagnostic workup took >1 year (time to diagnosis >1 year), we quantified the diagnostic delay.

### Statistical analysis.

The cumulative time-dependent probability of conversion to a TR state was calculated by the Kaplan-Meier estimate. The time of entry into the analysis was the date of diagnosis of epilepsy, and the time of the endpoint was the date of TR or the date of the last follow-up information (truncated at 40 years of follow-up), whichever came first. We performed univariate and multivariate Cox regression analyses to study the association between time to TR and prognostic factors. The results are presented as hazard ratios (HRs) and 95% confidence intervals (95% CIs). The assumption of proportional hazard was assessed by Schoenfeld residuals. Statistical analysis was performed with the Stata SE statistical package, version 14.0.^13^

### Standard protocol approvals, registrations, and patient consents.

The local ethics committee approved the study (10077).

## RESULTS

The final cohort included 139 patients (male/female 92/47) diagnosed with video-documented (57 patients, 41%) or video-EEG documented (82 patients, 59%) SHE (figure 1). The median follow-up time was 16 years (25th–75th percentile 7–25, 2,414 person-years).

One hundred ten patients were directly assessed between September 2012 and October 2013, while for 29 cases (2 deceased, 27 untraceable), we considered the seizure frequency at their last clinical assessment from medical records (median follow-up 11 years, 25th–75th percentile 5–23, 411 person-years, figure e-2).

The clinical features of our population are listed in table 1. The mean age at onset was 13.4 ± 10.2 years (range 1–56 years). The median time to diagnosis was 11 years (range 0–58 years, 25th–75th percentile 5–20 years). In 72 patients (53.7%, data missing in 5), there was a diagnostic delay of 12.8 ± 10.1 years (25th–75th percentile 2–59). Although not diagnosed with SHE at onset, another 37 patients (26.6%) received a diagnosis of focal epilepsy or the prescription of appropriate AEDs. Diagnostic delay was due to misdiagnosis (34), absence of medical counseling (16), or both (11), whereas in 11 cases, no specific causes were identified. Parasomnias represented the main reason for misdiagnosis (55.5%).

Sixty-three patients (45.3%) experienced at least one seizure in wakefulness during their history, while 76 patients (54.7%) showed typical SHE. Differences between the 2 groups are summarized in table e-1.

One hundred nineteen patients (85.6%) were sporadic cases. The remaining 20 (14.4%) reported a positive history for epilepsy within 2 degrees of relatedness. Seven of them (5.0%) belonged to 5 ADSHE pedigrees, while 13 (9.4%) had a family history for other focal epilepsies with or without SHE.

A neuroradiologic assessment was available in all but 4 patients. Brain MRI was performed in 128 patients; CT scan was performed in 7. Twenty-five patients with refractory SHE underwent a presurgical assessment, including stereo-EEG monitoring in 12 cases (table e-2).

Twenty-two (15.8%) were lesional cases. In 20 patients, gross/multiple abnormalities or focal cerebral lesions were detected on imaging (table e-2). Eight of the patients with focal lesions had MRI and neurophysiologic findings suggestive of focal cortical dysplasia (FCD) IIb, which was confirmed by histology in 2 cases. Additionally, in 2 patients with lesion-negative MRI who underwent surgery, FCD I and IIb were disclosed by histology.

Seven patients (5.4%) showed pathologic NE (table e-3). A formal neuropsychological evaluation, available in 73 cases, disclosed a variable degree of ID (IQ range 49–69) in 9 patients (12.3%) and borderline IQ in 7 (9.6%).

### Terminal remission.

Of the 139 patients, 43 (31%) were in remission at some point. Of these, 14 (10%) subsequently relapsed. The mean duration of the longest remission period was 9.14 ± 6.15 years (median 7, range 5–28 years).

**Figure 2:**
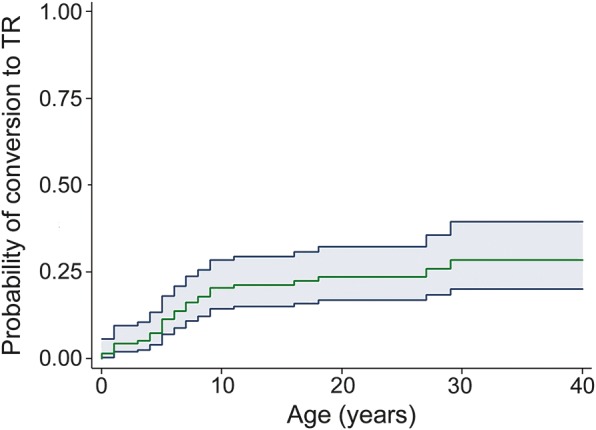
Terminal remission rate The green curve representing the cumulative probability of achieving terminal remission (TR) showed a slow trend of remission: 20.4% at 10 years from onset (97 patients at risk), 23.5% after 20 years (59 patients at risk), and 28.4% after 30 years (24 patients at risk) and after 40 years (13 patients at risk). The gray area represents the 95% confidence interval.

At the last assessment, 31 achieved TR (22.3%), and the probability for TR by 10, 20, and 30 years from inclusion was 20.4%, 23.5%, and 28.4%, respectively. The cumulative time-dependent probability of conversion to TR is shown in figure 2. Univariate association between time to TR and prognostic factors is reported in table 1.

The mean age at TR was 33.6 ± 16.75 years; the mean disease duration was 17.4 ± 13.46 years. At the last follow-up, 10 patients (32.3%) were still on antiepileptic treatment, and 21 (67.7%) were not taking any therapy. Among the latter, 13 (62%) withdrew AEDs after TR, according to medical prescription (one after surgery). Eight patients had a spontaneous TR: 3 patients (14.3%) discontinued therapy autonomously before attaining seizure control, while 5 patients (23.8%) had never taken AEDs.

At the last assessment, 108 of 139 patients failed to attain 5-year remission (NTR group 77.7%): 22 of them (20.4%) have had SF for <5 years, and 86 (79.6%) still have seizures.

Figure e-2 shows the outcome chart with patterns of seizure occurrence in TR and NTR patients.

Among the 86 NTR patients still with seizures, 20 (23.3%) were off medication: 5 had never started treatment, and 15 discontinued AEDs autonomously for ineffectiveness/severe side effects or because nocturnal events did not affect their quality of life. Most of them had a milder condition (lower seizure frequency, no seizures in wakefulness or bilateral tonic-clonic seizures except in one).

The remaining 66 NTR cases (76.7%) took adequate antiepileptic treatment without seizure control: 26 were on monotherapy, and 39 were taking a 2 to 5 AEDs (data not available in one). Of the 2 patients who died, one patient with obstructive sleep apnea comorbidity had a sudden unexpected death in epilepsy after 40 years of disease,^14^ while the other died of an event unrelated to epilepsy.

With the exclusion of possible factors of false drug resistance (such as poor compliance with AEDs), the overall rate of drug resistance was 38.8% (54 patients), ranging from degree IIa (5 patients) to IIIb (34 patients).

### Predictors of TR.

**Figure 3:**
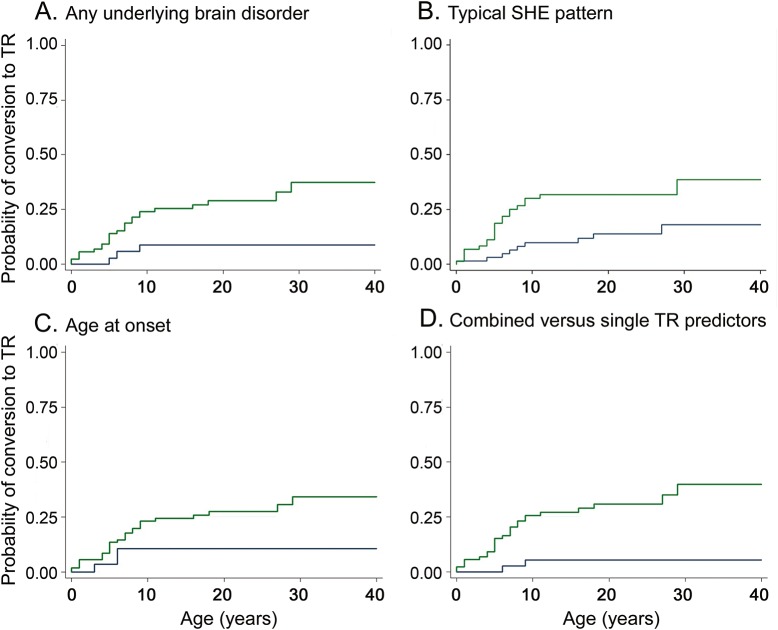
Terminal remission predictors Terminal remission (TR) as a function of (A) any underlying brain disorder (at least one of the following: intellectual disability, personal history of perinatal insult, pathologic neurologic examination, brain structural abnormalities): absence of any underlying brain disorder (green curve) and presence of any underlying brain disorder (blue curve) identified; (B) typical sleep-related hypermotor epilepsy (SHE; 100% of sleep-related events): patients with typical SHE (green curve) showed a higher TR rate than patients experiencing seizures in wakefulness (blue curve); (C) age at epilepsy onset: the cumulative probability of TR is higher in patients with age at onset ≥6 years (green curve) compared to patients with earlier epilepsy onset (blue curve); (D) combined vs single TR predictors (absence of any underlying brain disorder, typical SHE, and age at onset ≥6 years): the combination of at least 2 of the TR determinants identified (green curve) compared to having one or none of them (blue curve).

The cumulative time-dependent probability of conversion to TR by a single possible prognostic factor is reported in table 2. The absence of any underlying brain disorder (HR 4.21, 95% CI 1.26–14.05, *p* = 0.020, figure 3A) and typical SHE (HR 2.76, 95% CI 1.31–5.85, *p* = 0.008, figure 3B) were associated with TR. Age ≥6 years at onset showed a trend toward TR (HR 2.99, 95% CI 0.89–10.02, *p* = 0.076, figure 3C). The multivariate analysis model including these factors did not disclose any of them as statistically significant because they were strictly associated with each other (data not shown). However, their combination into one variable with 2 categories disclosed that patients with at least 2 of them (absence of any underlying brain disorder, typical SHE, age at onset ≥6 years) had an HR for TR of 7.38 (95% CI 1.75–31.21, *p* = 0.007) compared with patients having one or none of them (figure 3D). None of the patients with age at onset <6 years, with any underlying brain disorder, and not having typical SHE reached TR.

**Table 2:**
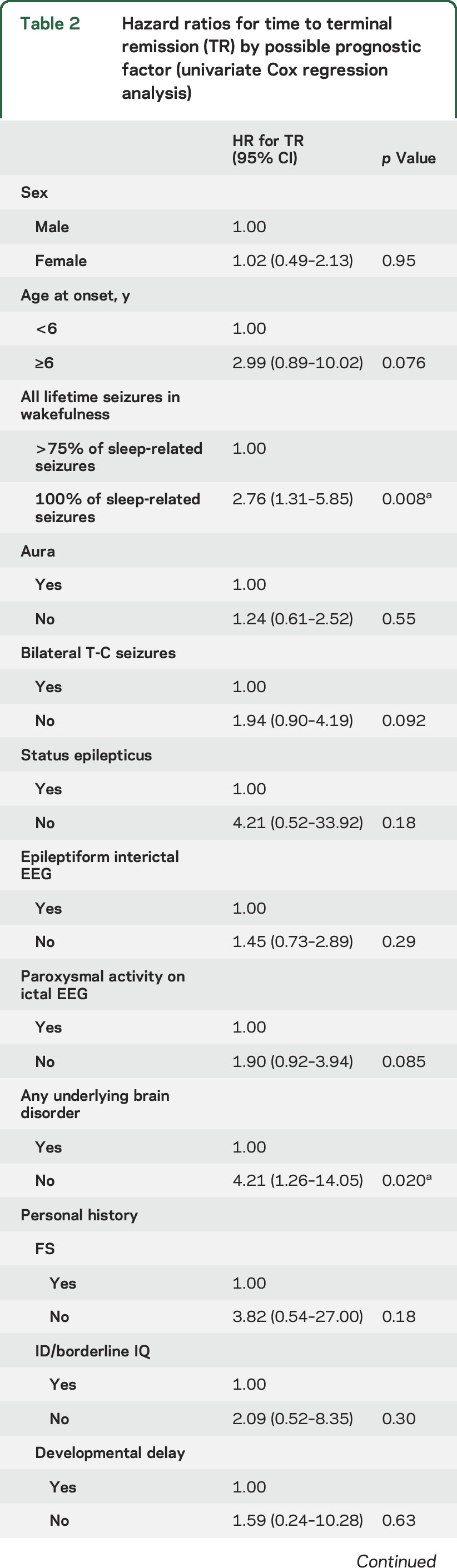

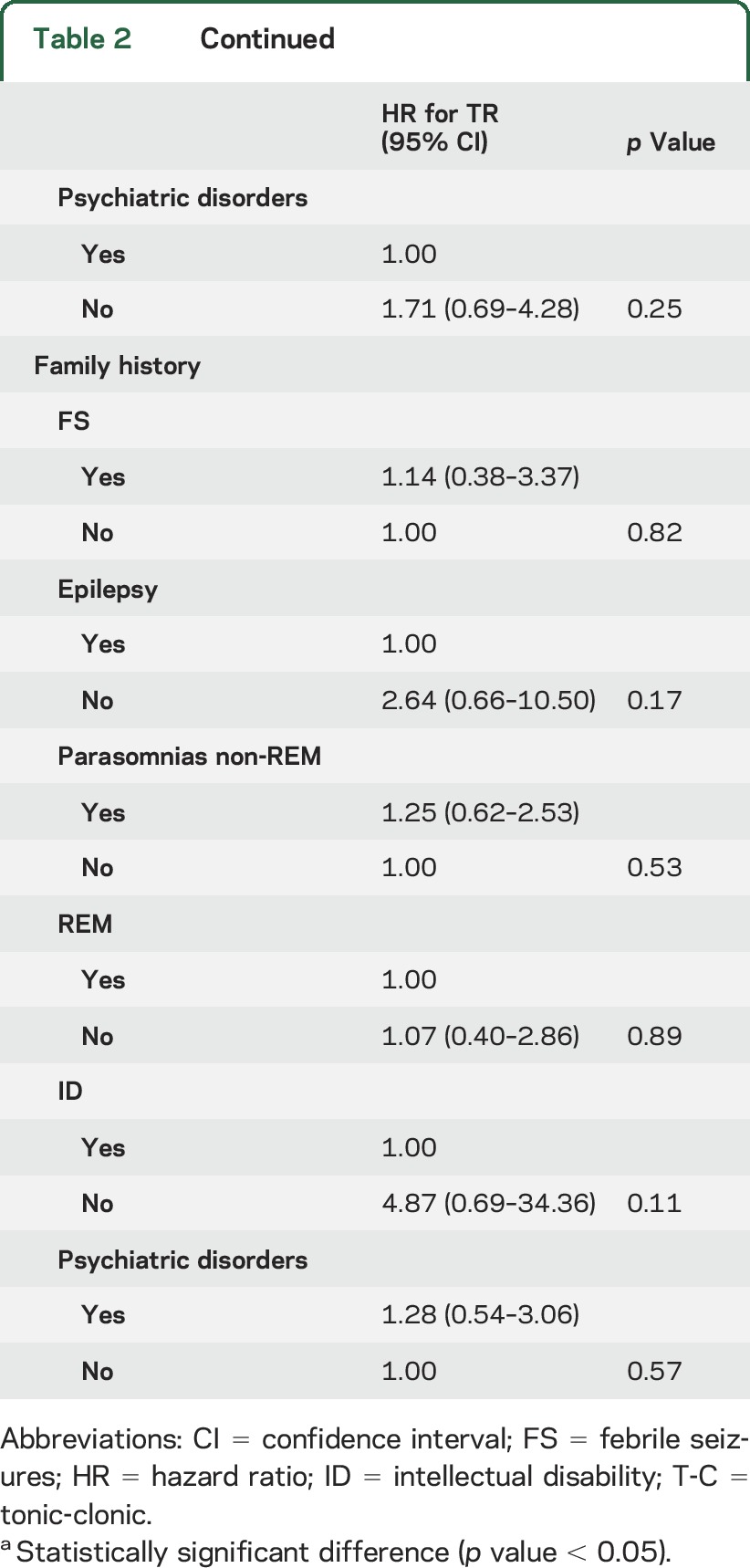
Hazard ratios for time to terminal remission (TR) by possible prognostic factor (univariate Cox regression analysis)

## DISCUSSION

We evaluated the prognostic features of a large, representative cohort of patients with SHE after a long-term follow-up. Most patients are sporadic cases of unknown etiology (70%), but we also included patients with brain lesions (16%) possibly associated with ID and abnormal NE (symptomatic/remote symptomatic etiology). Familial cases represent ≈14% of our cohort, confirming that inherited genetic SHE represents a restricted condition in the broad etiologic spectrum of this syndrome.^1,15^

Our study showed that only 22.3% of patients achieve TR after a median 16-year follow-up, most with a remitting pattern from disease onset (figure e-2). To date, studies on SHE prognosis have addressed overall surgical outcomes,^6,7^ with few reports available on nonsurgical case series.^5^ This precludes any comparison of our findings with other reports. Despite considerable differences in the definitions of remission, duration of follow-up, and patient selection, longitudinal cohort studies including mixed cohorts of new-onset epilepsies consistently report a remission rate of 65% to 85%,^16,17^ higher than that in our cohort. Focal epilepsies showed TR rates only somewhat less than that,^18–24^ although overall data on the possible importance of seizure type (focal vs generalized) in remission are controversial.^25^ Instead, the most important factor affecting remission appears to be symptomatic/remote symptomatic etiology vs nonsymptomatic etiology.^25,26^ Our data support this distinction, pointing to ID, perinatal insult, pathologic NE, and brain abnormalities in possible association (any underlying brain disorder) as a negative predictor of SHE outcome.

Moreover, age at onset showed a trend toward statistical significance; the lower the age at onset, the less likely it was for patients to enter TR. In previous studies on mixed cohorts of epilepsies, early age at onset has been reported as both a negative^23,27^ and a positive^19,28,29^ predictor. Because early-onset refractory SHE has been strongly associated with FCDs, particularly type IIb,^7,30^ the finding of early age at onset as a possible negative predictor in our cohort could suggest that an additional number of nonlesional cases may have an underlying structural etiology. Despite continuous improvements in MRI techniques, 15% of FCD type II is not detected by targeted conventional diagnostic imaging,^31^ as in 2 of our patients. The hypothesis that symptomatic cases due to subtle FCD are underestimated in our cohort could partly explain the low remission rate we found. Another factor affecting TR was the occurrence of seizures in wakefulness. Our cohort showed a higher percentage of seizure in wakefulness than other case series^2^ that occasionally reported daytime seizures, especially in periods of poor control. However, these studies did not address the lifelong incidence of seizures in wakefulness after such a long follow-up. In our study, comparison between these patients and the group with 100% of sleep-related events (typical SHE) disclosed significant differences in terms of epileptiform abnormalities, bilateral tonic-clonic seizures, status epilepticus, and variables suggestive of a symptomatic etiology (table e-1). Typical SHE resembles a milder form within the heterogeneous spectrum of the syndrome. Interestingly, patients belonging to this group also had a significantly more frequent family history of non-REM parasomnias, reinforcing the hypothesis of a common underlying mechanism (dysfunction of the arousal system) and suggesting a continuum with parasomnias.^32^

The NTR group encompasses a portion of patients with mild SHE declining long-term pharmacologic treatment because of either a poor recognition/acceptance of the diagnosis of epilepsy or the modest consequences of sleep-related seizures on quality of life, driving restrictions, and social stigmatization. We considered this a possible additional factor indirectly affecting the TR rate in our cohort. These patients were not taking AEDs and consequently were not drug resistant, which also explains why, despite the low remission rate, the drug resistance percentage we found (38.8%) differs little from that generally reported for epilepsy.^33^

The low TR rate in our cohort may also be related to the limits of a retrospective recruitment and to other methodological reasons, in particular our stringent definition of remission as 5-year SF. The rationale for this choice stemmed from evidence that briefer remissions are nonrobust indicators of long-term seizure outcome because many epilepsies follow a relapsing-remitting course.^10^ Moreover, we defined a relapse as the occurrence of even a single seizure after a 5-year remission, in contrast to other studies in which repeated seizures were necessary to define a relapse.^34^ It is also possible that our cohort overestimates the severity of SHE because, for diagnostic accuracy, we selected only patients with at least a hypermotor seizure documented on video-EEG, excluding milder cases with no seizures recorded (figure 1). Lastly, we noted a possible referral bias because the study was carried out in a tertiary center where patients with refractory epilepsy are assessed for presurgical study. However, the inclusion of patients with milder disease referred to the sleep center of our institute allowed us to achieve a population with a considerable variation in epilepsy severity. In addition, at the last follow-up, some patients were assessed by telephone interview to avoid the exclusion of patients who were diagnosed and treated initially at our center but did not return because of SF.

Our data show that SHE has a poor outcome after a long follow-up, pointing to a symptomatic/remote etiology as the most consistent determinant affecting its prognosis. Lesional cases could be underestimated in our cohort because of the limits of conventional neuroimaging in detecting subtle FCDs, for which surgery represents a highly effective treatment option.

## Supplementary Material

Data Supplement

## GLOSSARY

ADSHE: autosomal dominant sleep-related hypermotor epilepsy
AED: antiepileptic drug
CI: confidence interval
FCD: focal cortical dysplasia
HR: hazard ratio
ID: intellectual disability
NE: neurologic examination
NTR: not attaining terminal remission
SF: seizure freedom
SHE: sleep-related hypermotor epilepsy
STROBE: Strengthening the Reporting of Observational Studies in Epidemiology
TR: terminal remission
