# Supplementary material for: Sleep-related hypermotor epilepsy: Long-term outcome in a large cohort
Source: Neurology. 2017 Jan 3;88(1):70–7. doi: 10.1212/WNL.0000000000003459 (PMC5200852; doi:10.1212/WNL.0000000000003459)
Supplement: Data Supplement [file supp_88_1_70__index.html]

Sleep-related hypermotor epilepsy — Data Supplement 

# Sleep-related hypermotor epilepsy

## Data Supplement

**Neurology® data supplements are not copyedited before publication. Published editorials and translations have been copyedited.  
 © 2016 American Academy of Neurology.  
  
 Files in this Data Supplement:**

- e-Figures - PDF
- Appendix e-1 - Microsoft Word file
- Table e-1 - Microsoft Word file
- Table e-2 - Microsoft Word file
- Table e-3 - Microsoft Word file
