# Supplementary material for: Sleep-related hypermotor epilepsy: Long-term outcome in a large cohort
Source: Neurology. 2017 Jan 3;88(1):70–7. doi: 10.1212/WNL.0000000000003459 (PMC5200852; doi:10.1212/WNL.0000000000003459)

Figure e-1: Panel 1: clinical features and diagnostic criteria of SHE[1]

Panel 1: clinical features and diagnostic criteria of SHE[1]

Clinical features

Sleep-related Hypermotor seizures (HS):

- hyperkinetic features
  possible asymmetric tonic/dystonic posturing
- brief duration (<2 minutes)
- abrupt onset and offset
- stereotyped motor pattern

Occurrence of seizures in wakefulness, comorbidities with ID/neuropsychiatric disorders, absence of interictal and ictal scalp EEG abnormalities, extra-frontal origin of seizures do NOT represent exclusion criteria

Diagnostic criteria

Witnessed (possible) SHE
Clinical history of events consistent with HS, provided by an eye witness

Video-documented (clinical) SHE
Reliable audio-video documentation of HS:

- at least 1 event (preferably 2)
- clear visualization of the entire event
- event confirmed to be typical by witness

Video-EEG documented (confirmed) SHE
Video-EEG documentation of HS arising from sleep associated with:

- clear-cut epileptic discharge and/or
- interictal epileptiform abnormalities

Technical requirements:
- daytime sleep recording after sleep deprivation/full night sleep recording
- at least 19 EEG channels (10-20 International System)
- ECG, oculogram, chin EMG

Figure e-2: Outcome flow-chart.

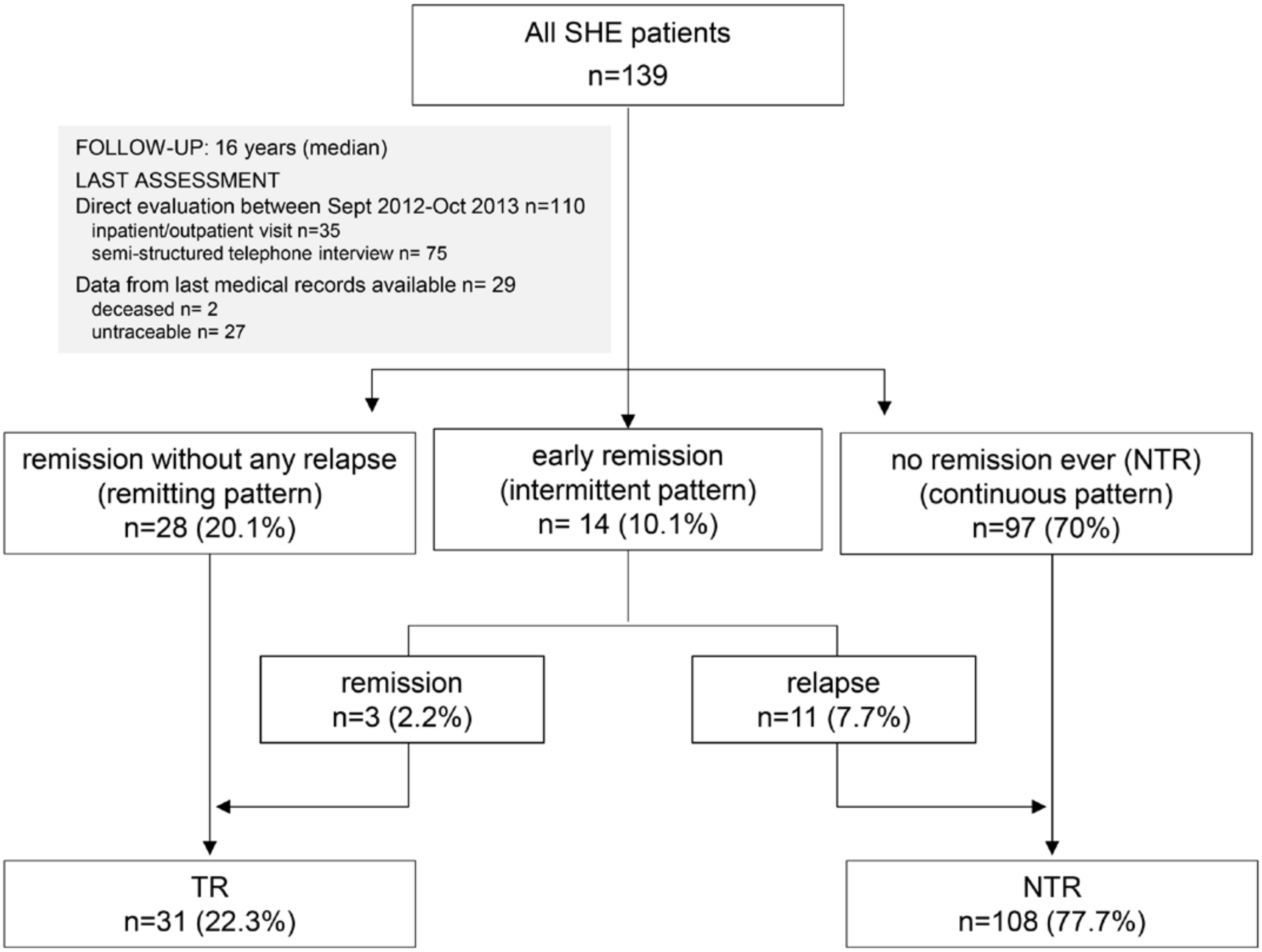

Supplement: Data Supplement [file supp_WNL.0000000000003459_e-figures.pdf]
