# Supplementary material for: Sleep-related hypermotor epilepsy: Long-term outcome in a large cohort
Source: Neurology. 2017 Jan 3;88(1):70–7. doi: 10.1212/WNL.0000000000003459 (PMC5200852; doi:10.1212/WNL.0000000000003459)
Supplement: Data Supplement [file supp_WNL.0000000000003459_Appendix_e-1.doc]

**SUPPLEMENTARY MATERIAL**

**Methods**

All consecutive patients referred to the Epilepsy and Sleep Centres at our Institute between 1980 and September 2012 for paroxysmal sleep-related events compatible with hypermotor seizures were reviewed. The final diagnosis of SHE was confirmed by three experts in sleep medicine and epileptology (PT, FP, FB). The agreement required was 100% otherwise cases were considered doubt and excluded.

At last assessment, clinical data were collected directly from each patient and whenever possible, from sleep witnesses.

**1. Details on database items and definitions**

1. *Personal data:* date of birth, gender and possibly exitus.
2. *Epileptic anamnesis* focused on age at epilepsy onset, type of seizures, presence of subjective sensation preceding motor manifestations, seizures during wakefulness, occurrence of bilateral tonic-clonic seizures and status epilepticus (SE), seizure frequency at onset and at our first and last assessments. We calculated diagnostic delay and disease duration.

Auras were classified according to the “Glossary of descriptive terminology for ictal semiology” of the ILAE Commission report.^e1^ Choking sensation or pharyngeal dysaesthetic auras were considered “viscera-sensory auras”.

To avoid overestimating the rate of seizures in wakefulness in patients who reported daytime seizures, we specifically investigated the co-occurrence of reduced vigilance.

Status epilepticus (focal or generalized), defined according to the Commission on Classification and Terminology of the International League against Epilepsy (1981),^e2^ was considered in particular when hospital admission and acute treatment with anticonvulsants was required.

Time to diagnosis was defined as the temporal gap between seizure onset and diagnosis.

Misdiagnosis was considered only when a clear-cut diagnostic mistake was made (e.g. “generalized idiopathic epilepsy”, “parasomnias”, “psychogenic non-epileptic seizures” or “panic attacks”). In these patients we carefully investigated the presence of atypical clinical features at epilepsy onset (e.g. seizures of different semeiology from typical nocturnal events or occurrence of seizures in wakefulness), as possible factors leading to misdiagnosis.

Disease duration, defined as the length of time seizures lasted, was calculated in all patients in terminal remission.

1. *Perinatal insult, personal history of febrile seizures (FS)*, *parasomnias, intellectual disability (ID) and psychiatric disorders, neurological examination (NE), obstructive sleep apnoea syndrome (OSA).*

Perinatal insult (i.e. hypoxic complication) was considered when prolonged hospitalization entailing incubation or assisted ventilation was documented by medical records. FS were defined according to the Commission on Epidemiology and Prognosis (1993).^e3^ Among NREM and REM-sleep parasomnias, we investigated confusional arousals, somnambulism, sleep terrors, REM behaviour disorder and nightmares, as particularly challenging to discriminate from sleep-related hypermotor seizures.

A formal neuropsychological evaluation including scales appropriate for age (WAIS/WAIR) was required to assess a borderline IQ (70-80) or presence of ID (IQ < 70). Psychiatric comorbidity was considered only when patients were diagnosed with a major psychiatric disorder requiring specific therapy. Similarly, OSA comorbidity was considered only with a polysomnographic diagnosis of OSA syndrome. Neurological evaluation (NE) was considered positive in case of gross abnormalities (e.g. hemiparesis) or multiple neurological deficits, possibly suggestive of syndromic cases. For patients who underwent surgery for epilepsy, we considered the pre-surgical NE.

1. *Family history of epilepsy, parasomnias, FS, ID and psychiatric disorders.*

Family history of epilepsy was assumed positive in case of recurrence of unprovoked epileptic seizures (sleep-related hypermotor seizures, other focal or generalised seizures) in relatives of first and second degree of kinship.

1. *Neurophysiological, neuroradiological and neurosurgical data*: we paid attention to interictal and ictal epileptiform abnormalities (specifying the distribution) and the sleep stage in which the episodes occurred. Neuroimaging was “positive” when MRI/CT showed both epileptogenic/potentially epileptogenic lesions and gross/multiple structural abnormalities. In case of non-specific lesions (e.g. white matter T2-hyperintensities), the examination was “unremarkable”. In a subgroup of patients with medically intractable epilepsy with unknown aetiology, negative MRI were reviewed by expert neuroradiologists for identification of subtle structural lesions. We collected data on pre-surgical assessment, possible SEEG, and neurosurgery, specifying the type of intervention and outcome according to Engel’s classification (Engel *et al*., 1993).^e4^
2. *Therapy, response to antiepileptic therapy and drug resistance.* We investigated past and current antiepileptic treatment, paying attention to doses of antiepileptic drugs, compliance and occurrence of possible idiosyncratic reactions.

Response to antiepileptic treatment was defined comparing seizure frequency at our first and last evaluation. We distinguished six possible groups: non-responders; patients with < 50% seizure reduction; patients with > 50% seizure reduction; SF on antiepileptic therapy; SF without AEDs (controlled withdrawal after SF period with medications); patients without therapy (autonomous withdrawal despite seizure recurrence or AEDs never started despite medical prescription).

**2. Statistical analysis**

Continuous variables are presented as mean ± standard deviation (SD), while categorical variables as absolute and relative frequency (%). Student’s t-test and Fisher’s exact test were used to compare variables among the TR group and NTR group. The unit of analysis was the patient. All p-values are based on 2-sided tests and p < 0.05 was considered significant.

**e-REFERENCES**

**e1** Blume WT, Luders HO, Mizrahi E, Tassinari C, van Emde Boas W, Engel J. Glossary of descriptive Terminology for ictal semiology: report of the ILAE task force on Classification and terminology. Epilepsia 2001; 42: 1212-8.

**e2** Commission on Classification and Terminology of the International League against Epilepsy. Proposal for revised clinical and electroencephalographic classification of epileptic seizures. Epilepsia 1981; 22: 489-501.

**e3** Commission on Epidemiology and Prognosis. International League Against Epilepsy. Guidelines for epidemiologic studies on epilepsy. Epilepsia 1993; 34: 592–595

**e4** Engel J Jr, Van Ness PC, Rasmussen TB, Ojemann LM. Outcome with respect to epileptic seizures. In: Engel J Jr, ed. Surgical treatment of the epilepsies. New York: Raven Press, 1993: 609- 21.
