# Supplementary material for: Sleep-related hypermotor epilepsy: Long-term outcome in a large cohort
Source: Neurology. 2017 Jan 3;88(1):70–7. doi: 10.1212/WNL.0000000000003459 (PMC5200852; doi:10.1212/WNL.0000000000003459)
Supplement: Data Supplement [file supp_WNL.0000000000003459_table_e-1.doc]

**Table e-1:** Electroclinical features of 76 patients with ”typical SHE”: comparison with patients also in wakefulness.

|  | | | **“Typical SHE”**  **(100% sleep-related seizures)** | | | **Seizures also in wakefulness**  **(75-99% sleep-related seizures)** | | | **P** |
| --- | --- | --- | --- | --- | --- | --- | --- | --- | --- |
|  | | | **Total**  **76** | **Valid % 54.7** | **Missing**  **(%)** | **Total**  **63** | **Valid %**  **45.3** | **Missing**  **(%)** |  |
| **Age at onset** | **< 6 years** | | 12 | 15.8 | - | 19 | 30.2 | - | 0.064 |
| **Frequency of**  **seizures at onset** | **Daily/multi-daily** | | 21 | 32.3 | 11 (14.5) | 31 | 54.4 | 6 (6.4) | 0.086 |
|  | **Weekly** | | 19 | 29.2 |  | 12 | 21.1 |  |  |
|  | **Monthly** | | 15 | 23.1 |  | 6 | 10.5 |  |  |
|  | **Yearly** | | 9 | 13.9 |  | 6 | 10.5 |  |  |
|  | **Sporadic** | | 1 | 1.5 |  | 2 | 3.5 |  |  |
| **Aura** | **Total** | | 20 | 26.3 | 1 (1.3) | 43 | 68.3 | - | **<0.001** |
| **Bilateral tonic-clonic seizures** | | | 10 | 13.2 | 2 (2.6) | 36 | 57.1 | 1 (1.6) | **<0.001** |
| **Status epilepticus** | | | 2 | 2.6 | 1 (1.3) | 12 | 19.1 | 0 (0.0) | **0.002** |
| **Interictal EEG** | **Epileptiform** | | 28 | 36.8 | - | 50 | 79.4 | - | **<0.001** |
|  | **Not specific** | | 41 | 53.9 | - | 50 | 79.4 | - | **<0.001** |
| **Ictal EEG abnormalities** | | | 25 | 32.9 | 3 (3.9) | 49 | 77.8 | 0 (0.0) | **<0.001** |
| **Pathological neurological evaluation** | | | 1 | 1.3 | 9 (11.8) | 6 | 9.5 | 1 (1.6) | **0.007** |
| **Abnormal neuroimaging** | | | 5 | 6.6 | 3 (3.9) | 15 | 23.8 | 2 (3.2) | **0.012** |
| **Any underlying brain disorder** | | | 8 | 10.5 | 10 (13.2) | 26 | 41.3 | 1 (1.6) | **<0.001** |
| **Personal history** | **FS** | | 4 | 5.3 | - | 9 | 14.3 | - | 0.084 |
|  | **Perinatal insult** | | 2 | 2.6 | 4 (5.3) | 6 | 9.5 | 32 (3.2) | 0.216 |
|  | **ID/ borderline IQ** | | 3 | 3.9 | - | 13 | 20.6 | - | **0.003** |
|  | **Developmental delay** | | 3 | 3.9 | 3 (3.9) | 4 | 6.3 | 1 (1.6) | 0.629 |
|  | **Psychiatric disorders** | | 16 | 21.1 | 7 (9.2) | 14 | 22.2 | 7 (11.1) | 0.895 |
|  | **OSA syndrome** | | 7 | 9.2 | 4 (5.3) | 5 | 7.9 | 5 (7.9) | 0.878 |
| **Family history** | **FS** | | 11 | 14.5 | - | 6 | 9.5 | - | 0.443 |
|  | **Epilepsy** | **Total** | 11 | 14.5 | - | 9 | 14.3 |  | 1.000 |
|  |  | **SHE** | 4 | 5.3 | - | 3 | 4.8 |  | 1.000 |
|  | **Parasomnias** | **NREM** | 39 | 51.3 | 1 (1.3) | 20 | 31.8 | 2 (3.2) | **0.042** |
|  |  | **REM** | 14 | 18.4 | 2 (2.6) | 9 | 14.3 | 2 (3.2) | 0.868 |
|  | **ID** | | 6 | 7.9 | 12 (15.8) | 9 | 14.3 | 15 (23.8) | 0.163 |
|  | **Psychiatric disorders** | | 16 | 21.1 | 14 (18.4) | 9 | 14.3 | 15 (23.8) | 0.547 |

**Abbreviation: FS**: febrile seizures; **ID**: intellectual disability; **OSA**: obstructive sleep apnoea.

Statistically significant differences (p value < 0.05) are in bold.
