# Supplementary material for: Sleep-related hypermotor epilepsy: Long-term outcome in a large cohort
Source: Neurology. 2017 Jan 3;88(1):70–7. doi: 10.1212/WNL.0000000000003459 (PMC5200852; doi:10.1212/WNL.0000000000003459)
Supplement: Data Supplement [file supp_WNL.0000000000003459_table_e-2.doc]

**Table e-2** Neuroradiological findings in 20 out of 139 SHE patient

|  |  | **type of lesion** | **n** | **localization** | **epileptiform eeg** | **seeg** | **neurosurgery** | **histology** | **engel class** |
| --- | --- | --- | --- | --- | --- | --- | --- | --- | --- |
| **FOCAL STRUCTURAL LESIONS** | **MCD** | FCD | 8 | l F | sharp W, l F | + | lesionectomy | FCD, IIb | Ia |
|  |  |  |  | l ant F | S-W, bil F-T > r | + | resection l T1\Heschl’s gyrus | FCD, IIb | Ib |
|  |  |  |  | l post T, Wernicke | sharp W/S-W, l T | + | - (excluded) | - | - |
|  |  |  |  | l insular-opercular | S-W, l F-T and C-T | + | - (excluded) | - | - |
|  |  |  |  | l dorsal-F | S, l F-C and T | - | - | - | - |
|  |  |  |  | r F | S-W, bil >l ant | - | - (refused) | - | - |
|  |  |  |  | l insular-opercular | S-W, bil F-C and T | - | - (excluded) | - | - |
|  |  |  |  | r gyrus cinguli | fast S, vertex and r F-C | - | - (refused) | - | - |
|  |  | Band heterotopia | 2 | r F | S-W, r F-T | - | - | - | - |
|  |  |  |  | l F inferior gyrus | S-W, l F-T | - | - (refused) | - | - |
|  |  | Micropolygyria + FCD | 1 | r F, ant- mesial | - | - | - | - | - |
|  | **OTHERS** | Vascular malformation (VM) | 1 | r F, paramedian | sharp W, r T-ant | + | internal corticectomy | Meningocortical VM | IIb |
|  |  | HS | 1 | l | S-W, bil T > l | - | - (not operable) | - | - |
|  |  | Hamartoma | 1 | l thalamus | Sharp W, r F-C | - | - | - | - |
|  |  | Cyst | 1 | l F basal | S-W, r F | - | - |  |  |
|  |  | Arachnoid cyst* | 1 | r T, sylvian fissure | - | - | - | - | - |
| **GROSS/ MULTIPLE**  **ABNORMALITIES** | | Diffuse leukoencephalopathy | 1 | bil periventricular | - | - | - | - | - |
|  |  | Post-traumatic lesions; cyst | 1 | l F; septum pellucidi | S/Sharp W, bil F-T > l | - | - | - | - |
|  |  | Hypoplastic CC, cereb hypotrophy* | 1 | splenium of CC | - | - | - | - | - |
|  |  | Hypoplasia + thick theca | 1 | r hemisphere | S-W, r F-T | - | - | - | - |

**Abbreviations:** **SEEG:** stereotactically implanted intracerebral electrodes; **MCD**: malformation of cortical development; **FCD**: focal cortical dysplasia; **HS**: hippocampal sclerosis; **cereb:** cerebellar; **CC:** corpus callosum**;**

**S:** spikes; **S-W**: spike-wave complex; **W:** wave; **F**: frontal; **T**: temporal; **C**: central; **P:** parietal; **O:** occipital; **l**: left; **r:** right; **bil**: bilateral; **>**: prevalent; **ant**: anterior; **post**: posterior; **Sup**: superior; **Inf**: inferior;

***** Only brain CT scan available.
