# Supplementary material for: Sleep-related hypermotor epilepsy: Long-term outcome in a large cohort
Source: Neurology. 2017 Jan 3;88(1):70–7. doi: 10.1212/WNL.0000000000003459 (PMC5200852; doi:10.1212/WNL.0000000000003459)
Supplement: Data Supplement [file supp_WNL.0000000000003459_table_e-3.doc]

| **Patient** | **Neurological evaluation** |
| --- | --- |
| 1 | Dysarthria and spastic tetraparesis |
| 2 | Right hemisomatic hypoplasia and abnormal osteotendinous reflexes |
| 3 | Severe papillary atrophy, bilateral upper limbs postural tremor, mild ataxia |
| 4 | Right-left confusion, finger agnosia, stuttering speech, dysdiachokinesia, bilateral negative myoclonus prevalent on the right, mirror movements |
| 5 | Right central facial paraesis, cognitive slowing, dysdiadocokinesia and impaired graphaesthesia |
| 6 | Bilateral hypoacusia, hyperexcitable blink reflex, frontal release signs |
| 7 | Scoliosis, excessive joint laxity, upper limbs hypotonia, abnormal posture |

**Table e-3** Neurological evaluation: pathological findings in 7 SHE patients.
